# The C-ETS2-TFEB Axis Promotes Neuron Survival under Oxidative Stress by Regulating Lysosome Activity

**DOI:** 10.1155/2016/4693703

**Published:** 2016-04-19

**Authors:** Shumin Ma, Zijun Fang, Wenwen Luo, Yunzhi Yang, Chenyao Wang, Qian Zhang, Huafei Wang, Huaiyong Chen, Chi bun Chan, Zhixue Liu

**Affiliations:** ^1^Key Laboratory of Nutrition and Metabolism, Institute for Nutritional Sciences, Shanghai Institutes for Biological Sciences, Chinese Academy of Sciences, University of Chinese Academy of Sciences, Shanghai 200031, China; ^2^Tianjin Haihe Hospital, Tianjin Institute of Respiratory Diseases, Tianjin 300350, China; ^3^The University of Oklahoma Health Sciences Center, 940 Stanton L. Young Boulevard, BMSB 634a, Oklahoma City, OK 73104, USA; ^4^School of Biological Sciences, The University of Hong Kong, 5N09, Kadoorie Biological Sciences Building, Pokfulam Road, Hong Kong

## Abstract

Excessive reactive oxygen species/reactive nitrogen species (ROS/RNS) produced as a result of ageing causes damage to macromolecules and organelles or leads to interference of cell signalling pathways, which in turn results in oxidative stress. Oxidative stress occurs in many neurodegenerative diseases (e.g., Parkinson's disease) and contributes to progressive neuronal loss. In this study, we show that cell apoptosis is induced by oxidative stress and that lysosomes play an important role in cell survival under oxidative stress. As a compensatory response to this stress, lysosomal genes were upregulated via induction of transcription factor EB (TFEB). In addition, localization of TFEB to the nucleus was increased by oxidative stress. We also confirmed that TFEB protects cells from oxidative stress both in vitro and in vivo. Finally, we found that C-ETS2 senses oxidative stress, activates TFEB transcription, and mediates the upregulation of lysosomal genes. Our results demonstrate a mechanistic pathway for inducing lysosomal activity during ageing and neurodegeneration.

## 1. Introduction

Oxidative stress, a concept of pathology that was first proposed by RS Sohal in 1990, occurs when an imbalance exists between the oxidant and antioxidant systems. In this case, excessive ROS cannot be eliminated by protective endogenous antioxidant pathways, and ROS gradually accumulate in vivo [1]. Additionally, some RNS have been shown to be produced and to accumulate under this condition [2]. Excessive ROS and RNS can result in DNA damage and protein and lipid modifications and can interfere with cell signalling pathways, eventually resulting in the generation of numerous diseases, including cancer, cardiovascular disease, and neurodegenerative disease [3].

Autophagy (meaning “self-eating” in Greek) is a highly conserved process found in yeast that is also used to maintain cellular homeostasis in higher eukaryotes, including humans. Autophagic processes degrade cellular macromolecules for energy use as well as clear nonessential or toxic proteins and damaged organelles [4]. Three forms of autophagy have been identified: chaperone-mediated autophagy, microautophagy, and macroautophagy. All three types use the same final pathway of lysosomal fusion and consequent substrate degradation [5]. Recently, more than 30 ATG (autophagy-related) proteins and approximately 50 lysosomal hydrolases have been identified [6]. Recent studies have shown that autophagy can be induced by oxidative stress, which is defined as a protective response that eliminates damaged cellular constitutes and prevents cell death [7]. It has been reported that starvation-induced ROS production can oxidize cysteine 81 of ATG4, leading to accumulation of this protein and autophagy activation [8]. Additionally, ROS can activate other proteins, such as I*κ*B kinase, NF*κ*B, sirtuins, and FOXO1, in the process of inducing autophagy. However, recent evidence suggests that RNS can also inhibit autophagy (e.g., NO can activate autophagy through inhibiting mTORC1). Other studies have shown that NOS overexpression suppresses autophagosome formation [5].

TFEB, a member of the MiT/TFE subfamily, has recently been identified as a major transcription factor of autophagy- and lysosome-related genes. TFEB overexpression in cultured cells significantly increases the number of autophagosomes and induces lysosomal biogenesis [9, 10]. It has also been reported that TFEB serine phosphorylation by extracellular signal-regulated kinase 2 regulates the location and activity of TFEB [10]. Another group reported that mTOR also phosphorylates TFEB at serine 211, causing retention of TFEB in the cytoplasm [11, 12]. Other studies have reported that TFEB-mediated autophagy helps to reduce protein aggregation while protecting neurons from neurotoxicity in the case of neurodegenerative diseases, such as Parkinson's disease and Huntington's disease [13, 14].

Several reports have shown that autophagy can be activated by oxidative stress and can act as a protective antioxidant pathway in cells. During the last stage of autophagy, lysosomes play a critical role in clearing macromolecules and organelles damaged by oxidative stress [4]. We speculate that lysosomes can also sense stress and that they play an important role in preventing stress-induced damage at the beginning. In this paper, we show that lysosomal activity is necessary for cell survival under oxidative stress and that lysosomal genes and TFEB are upregulated under oxidative stress. We further explore whether an increase in lysosomal genes under oxidative stress is regulated by TFEB and examine the transcription mechanism involved in this process, which may explain the upregulation of TFEB that is observed under oxidative stress.

## 2. Materials and Methods

### 2.1. Ethical Consent

Experiments involving isolation primary neurons and stereotaxic injections were performed in accordance with the Institute for Nutritional Science and national guidelines. This study was approved by the Animal Care Committee of the Institute for Nutritional Science.

### 2.2. Antibodies and Regents

Bafilomycin A1, chloroquine, N-acetyl-L-cysteine (NAC), and MPTP (1-methyl-4-phenyl-1,2,3,6-tetrahydropyridine hydrochloride) were purchased from Sigma-Aldrich; etoposide was purchased from Selleck. SS-31 was purchased from China Peptides. Ammonium chloride (NH_4_Cl) was purchased from Enox. Hydrogen peroxide was purchased from Shanghai Experiment Reagent Company. Anti-HA-Tag mouse monoclonal antibodies (HRP conjugate), -PARP (46D11) rabbit monoclonal antibodies, -caspase-3 rabbit polyclonal antibodies, -cathepsin D rabbit polyclonal antibodies, and -histone 3 (D1H2) rabbit monoclonal antibodies were purchased from Cell Signaling Technology. An anti-*α*-tubulin (ab80779) mouse monoclonal antibody and TFEB antibodies (ab113372) were purchased from Abcam. Anti-C-ETS2 (c-20) rabbit polyclonal antibodies were purchased from Santa Cruz Biotechnology, -LMNB1 (A2452) antibody was purchased from ABclonal, and -*β*-actin mouse monoclonal antibodies were purchased from Sigma-Aldrich. For immunohistochemical purposes, we used anti-tyrosine hydroxylase antibodies (T2928, Sigma) and -rabbit GFP antibodies (number 2956, Cell Signaling Technology).

### 2.3. Cell Culture and Treatment

The 293T cell line and SHY5Y human neuroblastoma cell line (purchased from ATCC) were cultured in DMEM supplemented with 10% FBS, 100 U/mL penicillin, and 100 U/mL streptomycin [15]. Primary hippocampal neurons were prepared from the hippocampi of neonatal mice (obtained from the SLRC Laboratory). We cut the hippocampi into pieces, digested them in 0.25% trypsin for 15 min at 37°C, and then washed them with a neurobasal medium with 10% FBS to stop trypsin activity. We then centrifuged and resuspended the cells in a neurobasal medium with 10% FBS and plated them onto polyethyleneimine-coated plates. After the cells were attached to the substrate, the medium was replaced with a neuronal culture medium (serum-free neurobasal medium with 2% B27, 0.5 mM glutamine, 100 U/mL penicillin, and 100 U/mL streptomycin) [16, 17]. The medium was replaced every two days. All of the cells were incubated in a humidified incubator with 5% CO_2_/95% air at 37°C.

The SHY5Y cells and primary neurons were treated with H_2_O_2_ at the indicated concentration for 4 h or were pretreated with bafilomycin A1 (50 *μ*M) [18], chloroquine (20 *μ*M) [19], and ammonium chloride (5 mM NH_4_Cl) for 1 h [20] and then exposed to H_2_O_2_ [17].

### 2.4. Plasmid DNA and RNA Interference

The human ETS2 gene was cloned into PCDNA3.1, with an HA tag and the human TFEB promoter, and the fragments were inserted into PGL3-basic vectors. Small-hairpin RNAs (shRNAs) targeting C-ETS2 (homo) and TFEB (homo) were cloned into PGE1 vectors for transfection. Adenoviruses containing shTFEB (Mus and Homo) and a pGIPZ lentivirus containing sh C-ETS2 (Mus) were packaged into MGH and 293T cells, respectively, for infecting SHY5Y cells and primary neurons, respectively. All of the shRNA sequences used are shown in Table 1.

### 2.5. Promoter Activity Assay

To study the responsiveness of TFEB to oxidative stress, we cloned the human* TFEB* promoter (1986 bp fragment upstream of the* TFEB* gene) and various fragments into the PGL3-basic luciferase reporter construct. After transfection with the indicated plasmids and Renilla luciferase plasmids as an internal control [21], a luciferase assay was performed with an assay kit obtained from Promega (Dual-Glo® Luciferase Assay System).

### 2.6. Western Blotting

Cells were lysed in an ice-cold lysis buffer of 50 mM Tris-HCl, pH 7.4, 150 mM NaCl, 5 mM EDTA, 1 mM PMSF, and complete protease inhibitor cocktail (Roche) for 15 min and then centrifuged at 12,000 rpm at 4°C for 15 min; the supernatant fraction was retained. Protein concentrations were quantified, and cell lysates were resolved by SDS-PAGE and used for immunoblotting. The proteins were electrophoresed on SDS-polyacrylamide gels and then transferred to a polyvinylidene fluoride membrane. The membranes were blocked in 5% skim milk in TBST (Tris, pH 7.4; 150 mM NaCl; and 0.1% Tween 20) for 1 h at room temperature and then incubated with the primary antibody at 4°C overnight [22]. The following antibody dilution levels were used: anti-TFEB (1 : 500); anti-PARP (1 : 1000); anti-caspase-3 (1 : 1000); anti-HA (1 : 1000); anti-cathepsin D (1 : 1000); and horseradish peroxidase-conjugated secondary IGG, anti-rabbit IGG, and anti-mouse IGG (1 : 7000).

### 2.7. Reverse Transcriptase-PCR Analysis

RNAiso Plus was used to isolate total RNA from 293T and SHY5Y cells and from mouse hippocampi. The RNA was measured spectrophotometrically based on the absorbance at 260 nm. One microgram of RNA was used as a template for quantitative reverse transcriptase- (RT-) PCR amplification using One Step SYBR® PrimeScrip*™* RT-PCR Kit (TaKaRa). Real-time PCR reactions were performed using an ABI 7900HT Real-Time PCR System with SYBR® Premix Ex Taq*™* II (TaKaRa). The relative abundance of transcripts was calculated based on normalization to the* GAPDH* gene. The primers are shown in Table 2.

### 2.8. Immunofluorescence Staining and Confocal Microscopy

SHY5Y cells were grown on glass coverslips for 24 h after treatment with H_2_O_2_. The cells were then washed with PBS, fixed with 4% paraformaldehyde (PFA) for 10–15 min, washed again with PBS, treated with 0.5% Triton-X 100 for 15 min, washed with PBS once again, and were finally permeabilized in a blocking buffer (3% BSA in PBS) for 1 h. Coverslips were then incubated with primary antibodies and with Alexa-488 conjugated secondary antibodies (Molecular Probes) for 1 h at room temperature and were then finally incubated in DAPI for 2 min. Coverslips were mounted on a glass slide with Fluoromount (Sigma), and images were obtained using a confocal microscope (Olympus FV1200).

### 2.9. Subcellular Fractionation

We removed the medium from plates, scraped off the cells, added ice-cold PBS, and spun the samples down at 3,000 rpm at 4°C. The supernatant was completely removed, added to 300 *μ*L of solution A on ice for 15 minutes, and then centrifuged at 3,000 rpm. The supernatant was saved as the cytosolic fraction. We then washed the medium twice with solution A, resuspended the pellet in solution B, mixed it and stored it on ice for 30 minutes. These samples were then centrifuged at 12,000 ×g for 10 minutes, and the supernatant was collected as the nuclear fraction. The protein levels of TFEB in the cytoplasm and nuclei were detected by western blotting. Solution A was composed of 10 mM HEPES (pH 7.9), 10 mM KCl, 2 mM MgCl_2_, 0.5 mM DTT, and 0.1% NP-40. Solution B was composed of 20 mM HEPES (pH 7.9), 0.2 mM EDTA, 1.5 mM MgCl_2_, 0.42 mM NaCl, 0.5 mM DTT, and 25% glycerol. For detection of phosphorylated TFEB in the nucleus and cytoplasm, samples were electrophoresed on SDS-polyacrylamide gel with Phos-tag and transferred to polyvinylidene fluoride membrane at 90 V overnight. The following procedures were performed according to the instructions in the Phos-tag*™* manual from Wako.

### 2.10. Annexin V Apoptosis Detection via Flow Cytometry

The apoptosis of primary neurons was detected via flow cytometry, and cells were harvested and washed with PBS. The following protocols were performed according to the procedures given in the Annexin V Apoptosis Detection Kit-APC (eBioscience, 88-8007-72).

### 2.11. TUNEL Staining

The SHY5Y cells were grown on glass for TUNEL staining. Apoptotic cells were identified by fluorescence microscopy according to the procedures given in the In Situ Cell Death Detection Kit (Roche, 12156792910).

### 2.12. Stereotaxic Injection of Adenovirus Viruses

Eight-week-old male C57BL/6 mice (obtained from SLRC Laboratory) were anesthetized and were then placed in a stereotaxic frame (purchased from Narishige Scientific Instrument Lab). A 1 *μ*L injection of adenovirus virus of GFP (1E + 11 PFU/mL) or TFEB (1E + 11 PFU/mL) was delivered unilaterally to the left substantia nigra pars compacta at a rate of 0.15 *μ*L/min. The coordinates for the mouse SCN were anterior-posterior −3.1 mm, medial-lateral −1.2 mm, and dorsal-ventral −4.6 mm, as measured from the bregma [23]. After surgery, the mice were warmed under a lamp until they awakened.

### 2.13. MPTP and Antioxidant Treatment

Eight-week-old male mice were intraperitoneally injected with MPTP (dissolved in 0.9% NaCl, 30 mg/kg body weight) or saline for five days at 24 h intervals. NAC (100 mg/kg) [24] and SS-31 (6 mg/kg) [25], both dissolved in PBS, were intraperitoneally injected into mice 30 mins before MPTP injection. Mice were killed 48 h after the last injection. For the stereotaxic injection group, mice were intraperitoneally injected with MPTP three days after virus delivery [26].

### 2.14. Immunohistochemistry

Mice were anesthetized and transcardially perfused first with PBS and then with 4% paraformaldehyde. Brains were removed, fixed in 4% paraformaldehyde for 1 h and then dehydrated in 30% sucrose overnight. In brief, 10 *μ*m thick cryosections were washed in PBS, fixed in 4% paraformaldehyde, and permeabilized in 0.5% Triton-X 100 in PBS. Sections were blocked in 3% normal goat serum in PBS and were incubated with primary antibodies overnight at room temperature (anti-tyrosine hydroxylase (1 : 200) and anti-GFP (1 : 100)). After washing, sections were incubated with fluorescently labelled secondary antibodies (A11004 and A11008, Invitrogen) for 2 h at room temperature. After being washed, sections were cover-slipped with Fluoromount (F-4680, Sigma).

### 2.15. TH Neuron Quantification

Dopaminergic neurons were detected via immunohistochemistry using tyrosine hydroxylase antibodies. TH-positive cells in the SN were counted in a single-blind manner using ImageJ software. Six sections (under the microscope, 10x) from five mice were analysed [27].

### 2.16. Statistical Analysis

A statistical analysis was carried out with GraphPad Prism 5.0 software. The quantitative data are expressed as the mean ± SEM. Statistical significance was determined through a Student *t*-test or one-way ANOVA. *P* < 0.05 was considered statistically significant.

## 3. Results

### 3.1. Lysosomal Functions Protect Cells from Oxidative Stress-Induced Cell Apoptosis

**Figure 1:**
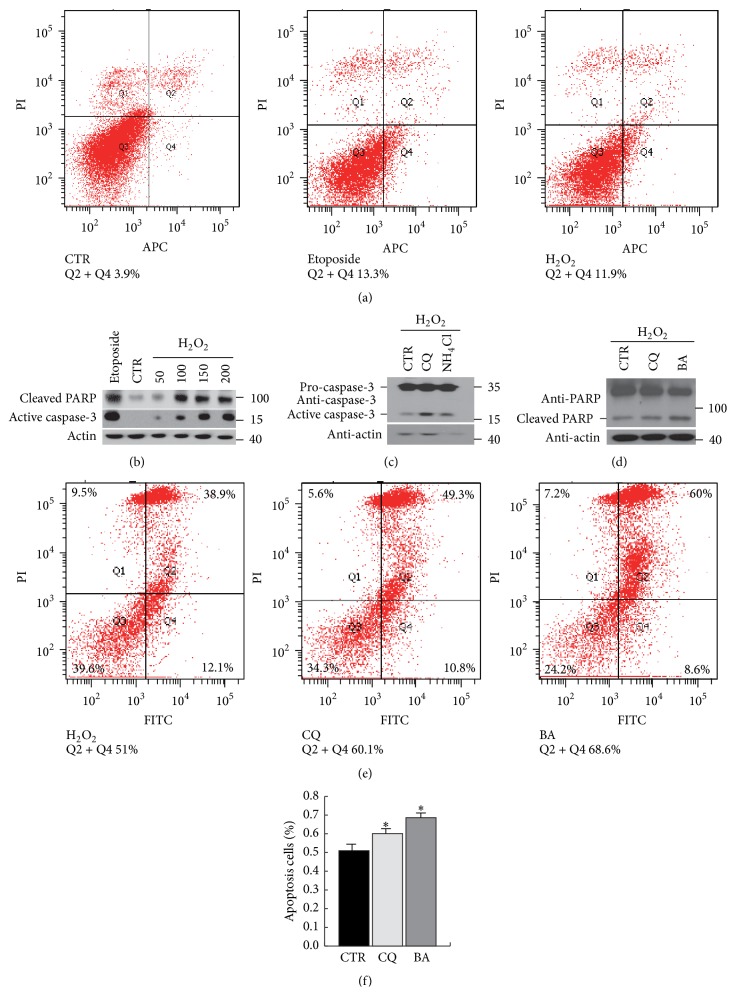
Lysosome functions are critical for cell survival under oxidative stress. (a) Primary neurons were treated with H_2_O, H_2_O_2_ (50 *μ*M), or etoposide (80 *μ*g/mL) as a positive control for 4 h, and cell apoptosis was detected by flow cytometry. (b) Primary neurons were treated with different concentrations of hydrogen peroxide or etoposide for 4 h, and cell apoptosis was detected by immunoblotting with PARP and caspase-3 antibodies. (c, d, e, f) Primary neurons were pretreated with the lysosome inhibitor bafilomycin A1, chloroquine, or ammonium chloride for 1 h and then exposed to H_2_O_2_ (150 *μ*M) for 4 h. The degree of apoptosis was analysed by western blotting or flow cytometry. The flow cytometry data were quantified, and the results are shown in (f). Values are the means ± SEM. ^*∗*^ *P* < 0.05 compared with the control group.

Neurons in the brain have been found to be more vulnerable to oxidative stress because of their high levels of oxygen utilization and ATP synthesis. Excessive ROS-induced damage to proteins, lipids, DNA, and organelles can result in cell death, which is a common feature of many neurodegenerative diseases [4]. To confirm whether oxidative stress can induce cell apoptosis, we treated primary neurons with hydrogen peroxide. Flow cytometry data show that cell apoptosis can be induced by hydrogen peroxide (Figure 1(a)). A western blot analysis of cleaved PARP (poly(ADP-ribose) polymerase) and active caspase-3 indicated an increase in apoptosis levels with increasing hydrogen peroxide doses (Figure 1(b)). As the terminal sites of macromolecular degradation, lysosomes can clear oxidative products and help cells maintain intracellular homeostasis. Next, we explored the role of lysosomes in cell survival under oxidative stress. Primary neurons were pretreated with the lysosome inhibitors CQ, BA, or NH_4_Cl and then exposed to hydrogen peroxide. Cell apoptosis was detected by western blot analysis and flow cytometry (Figures 1(c)–1(f)). We found that lysosome inhibition increased the cell apoptosis induced by oxidative stress. These results show that lysosomal functions are essential for cell survival under oxidative stress.

### 3.2. Oxidative Stress-Induced Expression of Lysosomal Genes

**Figure 2:**
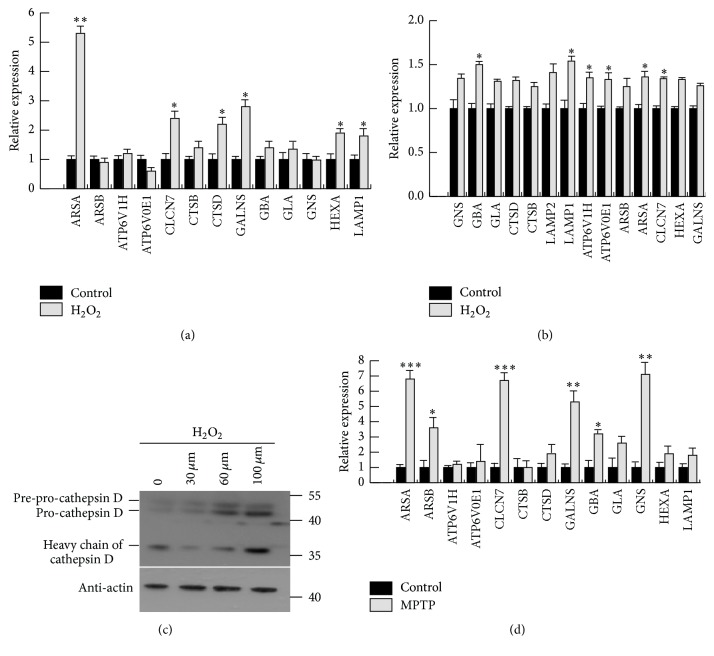
Oxidative stress-induced lysosomal genes expression. (a, b) SHY5Y cells or primary neurons were treated with H_2_O_2_ for 4 h and lysosomal gene levels were analysed by RT-PCR. Values are the means ± SEM. ^*∗*^ *P* < 0.05 compared with the H_2_O group. (c) SHY5Y cells were treated with different concentrations of H_2_O_2_ and the lysosomal enzyme cathepsin D was detected by western blot analysis. (d) Lysosomal genes of brains treated with or without MPTP were analysed by RT-PCR. Values are the means ± SEM. ^*∗*^ *P* < 0.05, ^*∗∗*^ *P* < 0.01, and ^*∗∗∗*^ *P* < 0.001 compared with the saline group.

It has been found that lysosomal activity is enhanced by protein deposition in the hippocampus in the case of early-stage Alzheimer's disease and that lysosomal activation promotes the clearance of the accumulated proteins and restores microtubule integrity [28]. To investigate lysosome function under oxidative stress, we analysed lysosomal gene expression in SHY5Y cells and primary neurons under oxidative stress. RT-PCR data show that lysosomal gene levels increased significantly as a result of oxidative stress relative to control levels (Figures 2(a) and 2(b)). The levels of the proenzyme and activated forms of cathepsin D also increased with increasing hydrogen peroxide concentrations (Figure 2(c)). These data show that the mRNA and protein levels of lysosomal genes were both increased under oxidative stress and that enzyme activities also increased. MPTP (1-methyl-4-phenyl-1,2,3,6-tetrahydropyridine), a neurotoxin that causes permanent symptoms of Parkinson's disease by destroying dopaminergic neurons in the substantia nigra of the brain, increases ROS levels, and this compound has been used to evaluate disease models in various animal studies. We analysed mRNA levels in the brains of MPTP treatment mice and found that lysosomal gene levels were markedly increased relative to those of the saline group (Figure 2(d)). These data show that ROS can cause increased lysosomal gene expression in vivo and in vitro.

### 3.3. TFEB Increased and Mediated the Upregulation of Lysosomal Genes under Oxidative Stress

**Figure 3:**
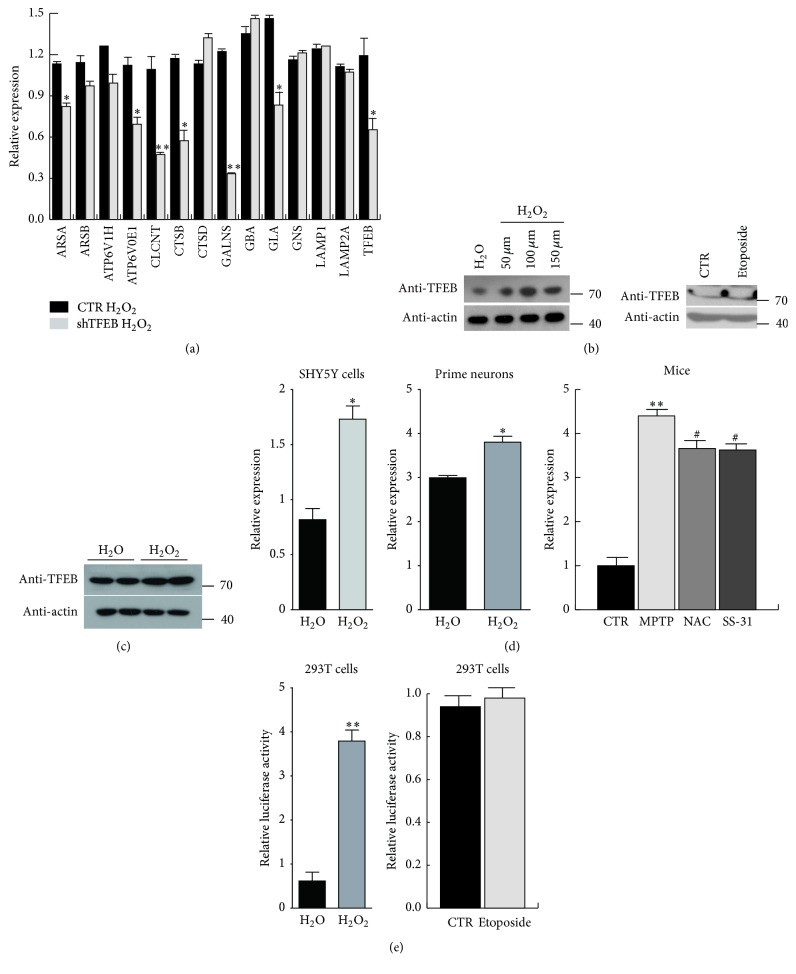
The increased expression of lysosomal genes is regulated by TFEB under oxidative stress. (a) 293T cells were transfected with PGE1-shTFEB plasmids or PGE1 empty plasmids as a control and, after 24 h, were treated with H_2_O_2_ for 4 h. Lysosomal genes were analysed by RT-PCR. Values are the means ± SEM. ^*∗*^ *P* < 0.05, ^*∗∗*^ *P* < 0.01 compared with the empty vector group. (b) SHY5Y cells were treated with H_2_O_2_ or etoposide, and the protein level of TFEB was detected. (c) Primary neurons were treated with H_2_O_2_, and the protein level of TFEB was detected. (d) The mRNA level of TFEB in SHY5Y cells, primary neurons, and mouse brains was measured using RT-PCR. Values are the means ± SEM. ^*∗*^ *P* < 0.05, ^*∗∗*^ *P* < 0.01 compared with the control group, and ^#^ *P* < 0.05 compared with the MPTP group. (e) 293T cells were transfected with PGL3 constructs containing the* TFEB* promoter and the Renilla plasmid, and a luciferase assay was performed to measure the transcription activity of TFEB in the presence of H_2_O_2_ or etoposide. Values are the means ± SEM. ^*∗∗*^ *P* < 0.01 compared with the H_2_O group.

Recent studies have reported that TFEB constitutes a key transcription factor of lysosomal biogenesis. As overexpression of TFEB promotes lysosomal gene expression and that it accelerates the clearance of accumulated proteins [9], we speculated that TFEB may be involved in the upregulation of lysosomal genes under oxidative stress. Thus, we knocked down TFEB by transfecting plasmids containing TFEB-shRNA into 293T cells and measured the mRNA levels of lysosomal genes under oxidative stress. The results show that the expression of most lysosomal genes declined relative to that of the control (Figure 3(a)). To explore whether TFEB is regulated by oxidative stress, we determined the protein and mRNA levels of TFEB. First, the protein levels of TFEB were measured under oxidative stress. Figures 3(b) and 3(c) show that TFEB protein levels increased in SHY5Y cells and primary neurons after hydrogen peroxide treatment. To exclude the possibility that TFEB upregulation was induced by apoptosis, we treated SHY5Y cells with etoposide and found no changes in TFEB protein levels compared with control levels. The TFEB mRNA levels were also elevated in SHY5Y cells and primary neurons under oxidative stress, as determined by RT-PCR analysis. We then confirmed the results in mice. MPTP, which has been reported to cause oxidative stress in the brains of mice, induced TFEB upregulation in the animals, whereas the antioxidants NAC and SS-31 significantly reduced TFEB expression (Figure 3(d)). To determine whether the upregulation of TFEB occurred at the transcriptional level, we transfected a PGL3 construct containing the TFEB promoter into 293T cells and measured luciferase activity. As expected, hydrogen peroxide activated TFEB transcription. In addition, etoposide did not result in increased TFEB activation (Figure 3(e)). These results suggest that TFEB expression may be activated by hydrogen peroxide and that it is involved in the increased expression of lysosomal genes.

### 3.4. TFEB Translocated into the Nucleus under Oxidative Stress

**Figure 4:**
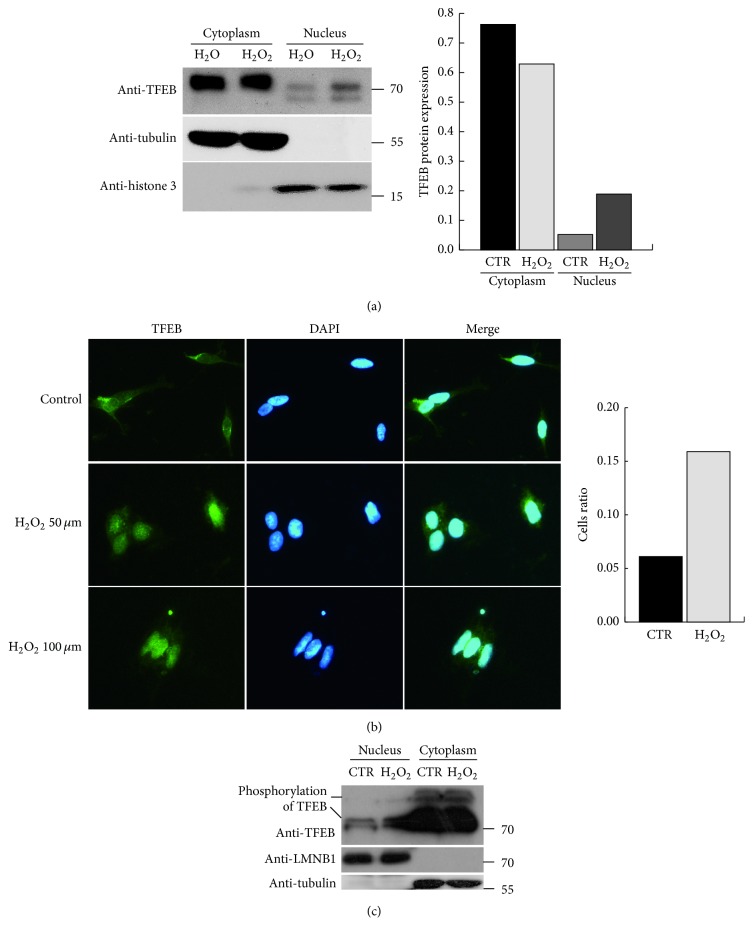
TFEB translocated to the nucleus under oxidative stress. (a) SHY5Y cells were treated with H_2_O or H_2_O_2_ and the subcellular fraction was separated to analyse the levels of TFEB proteins in the cytoplasm and nucleus. The histogram on the right shows the quantification of the western blot results. (b) The localization of TFEB was detected by immunofluorescence staining and observed via confocal microscopy. The histogram on the right shows percentage of cells in which TFEB was found in the nucleus. (c) SHY5Y cells were treated with H_2_O or H_2_O_2_, and the subcellular fraction was obtained. These protein samples were electrophoresed on SDS-polyacrylamide gels with Phos-tag to enable observation of the phosphorylation status of TFEB.

As our previous data show, downstream genes of TFEB were upregulated by hydrogen peroxide. We speculate that, as a transcription factor, TFEB may translocate to the nucleus to initiate lysosomal gene expression under oxidative stress. A subcellular fraction sample was obtained from SHY5Y cells, and protein levels of TFEB were analysed by western blotting. The data show that TFEB protein levels in the nucleus increased after treatment with hydrogen peroxide (Figure 4(a)). This result was also found following the immunofluorescence staining for TFEB in SHY5Y cells (Figure 4(b)). TFEB was mostly found in the cytoplasm under normal conditions. Although a significant amount of TFEB was present in the nucleus after incubation with hydrogen peroxide, the majority was still distributed in the cytoplasm. It has been reported that TFEB translocation is affected by its phosphorylation state. Therefore, we investigated the phosphorylation level of TFEB in the nucleus. The results show that the phosphorylation of TFEB also increased in the nucleus, which was likely due to the increased level of TFEB protein (Figure 4(c)).

### 3.5. TFEB Plays an Important Role in Cell Survival under Oxidative Stress

**Figure 5:**
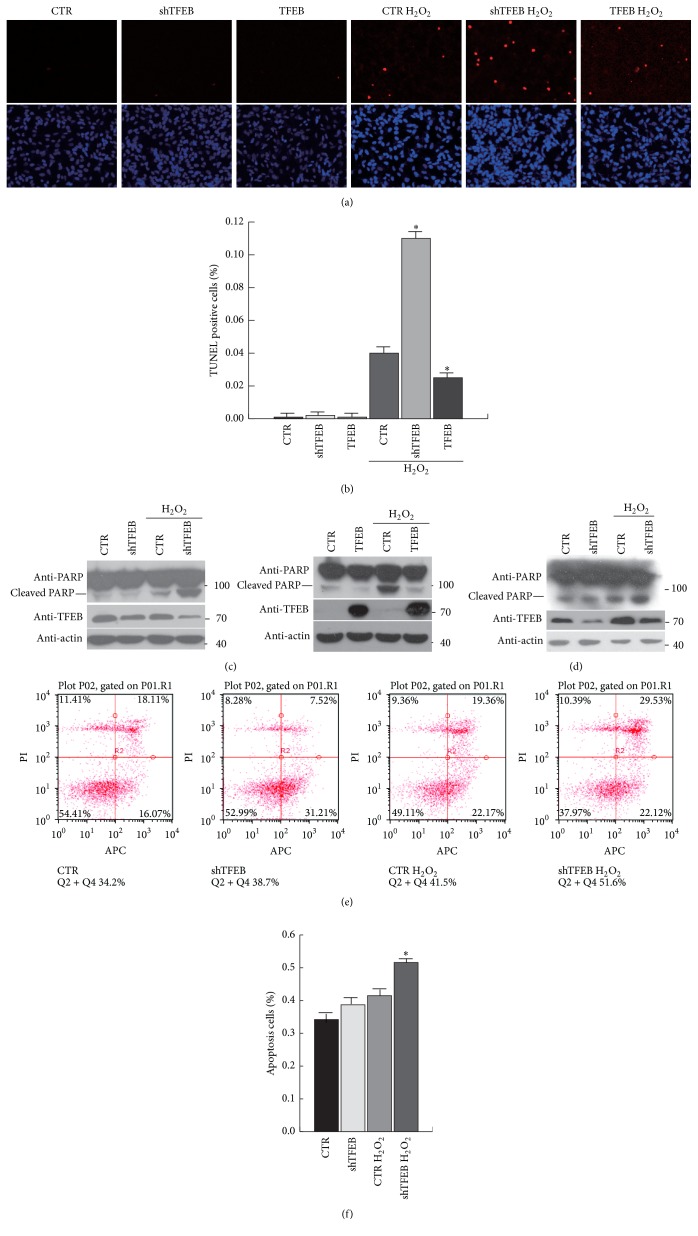
TFEB protects cells from apoptosis under oxidative stress. (a, b, c) SHY5Y cells were infected with a TFEB-knockdown or a TFEB-overexpression adenovirus for 48 h and then treated with H_2_O_2_ (100 *μ*M) for 12 h (for TUNEL staining) or 4 h (for western blotting). Apoptosis was detected by TUNEL staining and western blot analysis. The proportion of apoptotic cells was counted using ImageJ software, and 3–6 fields of each cover slide were analysed. The results are shown in (b). (d, e, f) Primary neurons were infected with a TFEB-knockdown adenovirus for 48 h and treated with H_2_O_2_ (150 *μ*M) for 4 h. Apoptosis was detected by western blot analysis and flow cytometry. (f) shows the flow cytometry data. Values are the means ± SEM. ^*∗*^ *P* < 0.05 compared with the control group.

We found that TFEB mediates the upregulation of lysosomal genes under oxidative stress, which led us to further explore how TFEB contributes to cell survival under oxidative stress. shTFEB was used for knockdown of TFEB expression in SHY5Y cells. Cell apoptosis was assessed with and without H_2_O_2_ treatment via TUNEL staining (Figures 5(a) and 5(b)). The results show that apoptosis was distinctly increased in TFEB KD cells relative to control cells exposed to hydrogen peroxide and that TFEB knockdown could not induce apoptosis under normal conditions. Conversely, TFEB overexpression in SHY5Y cells reduced apoptosis under oxidative stress. Cell apoptosis was also determined by western blotting, and the results were consistent with the TUNEL staining results (Figure 5(c)). We confirmed these results in primary neurons. The western blot analysis and flow cytometry results show that apoptosis increased following TFEB knockdown under oxidative stress (Figures 5(d), 5(e), and 5(f)). All of these results show that TFEB plays a key role in cell survival in response to oxidative stress.

### 3.6. TFEB Overexpression Protects Dopaminergic Neurons in the SN under MPTP-Induced Oxidative Stress

**Figure 6:**
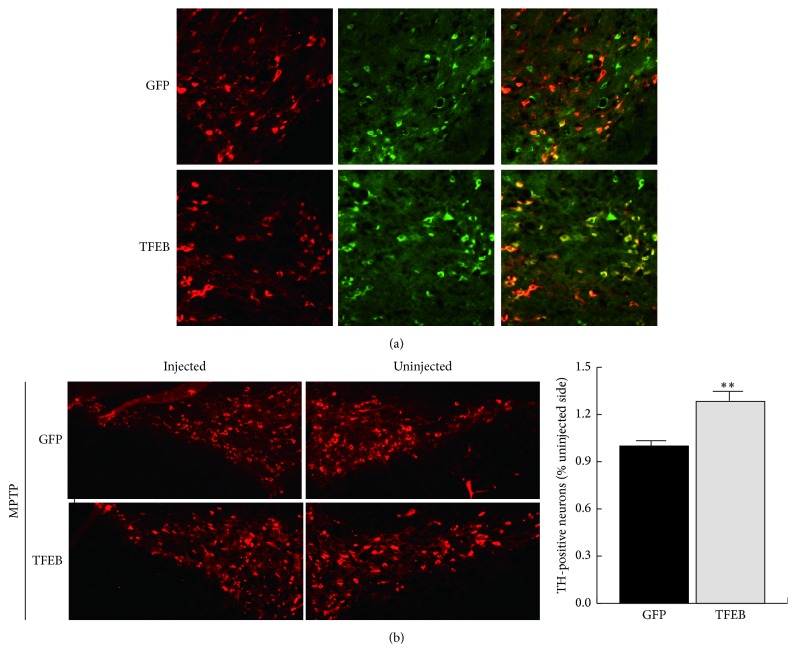
Overexpression of TFEB rescues dopaminergic neurons of the SN in MPTP-treated mice. (a) GFP or TFEB adenovirus expression in the SN is shown in the figure. Cryosections were immunostained for GFP (green) or tyrosine hydroxylase (red). (b) TH immunohistochemistry showing DA neurons in the GFP and TFEB groups. The numbers of TH-positive neurons were compared with those on the uninjected side. The quantified data are shown in the histogram. Values are the means ± SEM. ^*∗∗*^ *P* < 0.01 compared with the GFP group.

In this study, we induced oxidative stress in the SN by intraperitoneally injecting MPTP. It has been reported that MPTP can produce severe symptoms such as those observed with PD decline (e.g., impaired ATP production, loss of mitochondrial membrane potential, and ROS formation) [26]. We stereotaxically injected GFP and TFEB viruses in the SN before injecting MPTP and analysed TH-positive neurons in the midbrain. The corresponding viral expression results are shown in Figure 6(a). We then immunostained for tyrosine hydroxylase and assessed the number of TH-positive neurons in the SN. We found no differences in green fluorescence in mice between the injected and uninjected sides. However, TFEB overexpression in the SN significantly reduced the levels of neuronal loss relative to those found in the uninjected sides (Figure 6(b)).

### 3.7. C-ETS2 Regulates TFEB Expression under Oxidative Stress

**Figure 7:**
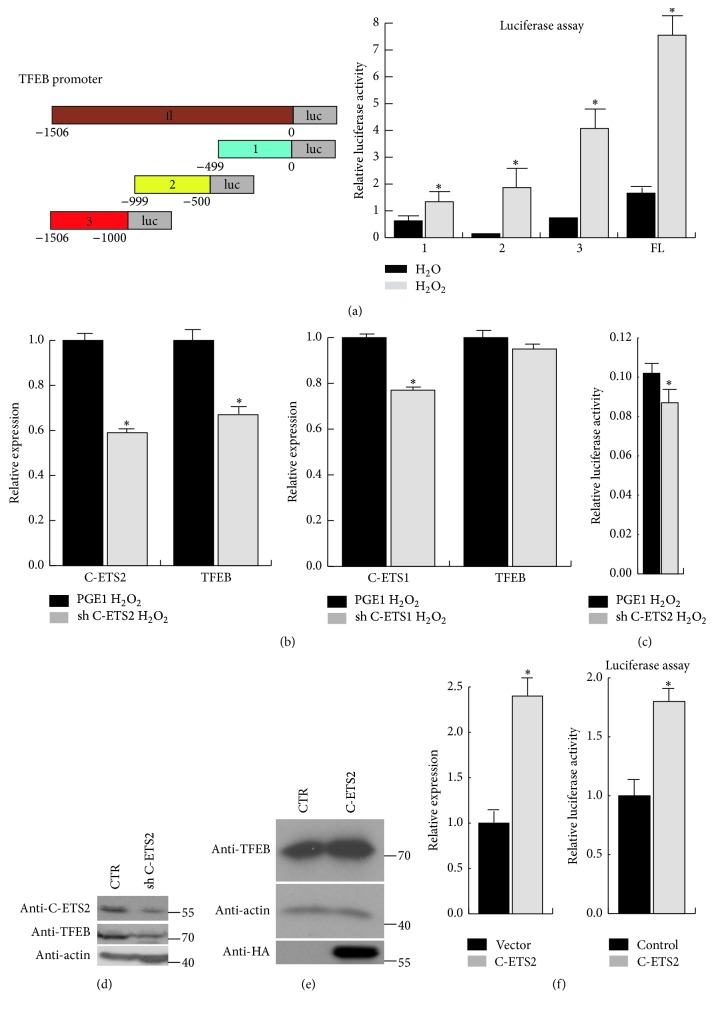
TFEB upregulation is controlled by C-ETS2 under oxidative stress. (a) TFEB promoter was truncated into three fragments and the luciferase activity of each fragment was assessed in 293T cells after treatment with H_2_O_2_ via a luciferase assay. Values are the means ± SEM. ^*∗*^ *P* < 0.05 compared with the H_2_O group. (b) C-ETS2 or C-ETS1 knockdown plasmids were transfected into 293T cells, and the transcription activity of TFEB was analysed by luciferase assay. Values are the means ± SEM. ^*∗*^ *P* < 0.05 compared with the empty vector group. (c, d) C-ETS2 knockdown plasmids were transfected into 293T cells, and the TFEB mRNA and protein levels were measured by luciferase assay and western blot analysis. (e, f) TFEB expression was analysed in 293T cells overexpressing HA-*C-ETS2* via western blot, RT-PCR, and luciferase assays. Values are the means ± SEM. ^*∗*^ *P* < 0.05 compared with the empty vector group.

We have demonstrated that TFEB transcription is activated by oxidative stress. To determine which transcription factor is involved in TFEB transactivation under oxidative stress, we randomly truncated the TFEB promoter into three fragments and performed a luciferase assay in 293T cells. The results show that the levels of all of the fragments were markedly increased under oxidative stress (Figure 7(a)). Then, we examined possible transcriptional factors for each of the three fragments on the PROMO website and found several candidate transcription factors that the fragments have in common (e.g., C-ETS1, C-ETS2, XBP-1, E47, and C/EBP*β*). We reviewed the literature and found that C-ETS1 and C-ETS2 are upregulated in response to oxidative stress [22, 29]. Thus, we speculated that C-ETS1 and C-ETS2 may be correlated with TFEB expression under oxidative stress. Next, we knocked down C-ETS1 and C-ETS2 in 293T cells and examined the expression of TFEB via RT-PCR. The results show that TFEB expression may be affected by C-ETS2 rather than by C-ETS1 (Figure 7(b)). We then further analysed TFEB expression via a luciferase assay and western blotting, with both showing that TFEB is regulated by C-ETS2 (Figures 7(c) and 7(d)). We then overexpressed C-ETS2 in 293T cells and detected TFEB expression. Based on the western blot, RT-PCR, and luciferase assay results, we conclude that C-ETS2 regulates TFEB expression under oxidative stress (Figures 7(e) and 7(f)).

### 3.8. C-ETS2 Is Involved in the Transcriptional Activation of Lysosomal Genes under Oxidative Stress

**Figure 8:**
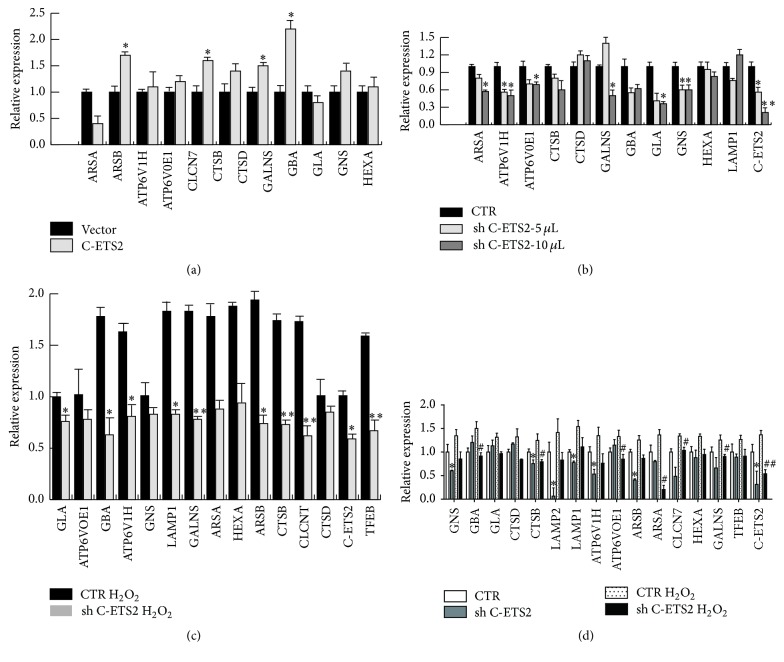
C-ETS2 mediates the expression of lysosomal genes induced by oxidative stress. (a) C-ETS2 was overexpressed in 293T cells and lysosomal genes were detected via RT-PCR. Values are the means ± SEM. ^*∗*^ *P* < 0.05 compared with the empty vector group. (b) 293T cells were infected with different amounts of the C-ETS2-knockdown adenovirus, and lysosomal genes were detected via RT-PCR. Values are the means ± SEM. ^*∗*^ *P* < 0.05, ^*∗∗*^ *P* < 0.01 compared with the GFP virus group. (c, d) SHY5Y cells and primary neurons were infected with the C-ETS2-knockdown adenovirus and the lentivirus, respectively, under oxidative stress and lysosomal genes were detected via RT-PCR. Values are the means ± SEM. ^*∗*^ *P* < 0.05, ^*∗∗*^ *P* < 0.01 compared with the GFP virus group in (c); ^*∗*^ *P* < 0.05, ^#^ *P* < 0.05, and ^##^ *P* < 0.01 compared with the control lentivirus group after H_2_O or H_2_O_2_ treatment, respectively, in (d).

The data described above show that C-ETS2 regulates TFEB expression under oxidative stress. To confirm that C-ETS2 is indeed involved in the upregulation of lysosomal genes in response to oxidative stress, we overexpressed C-ETS2 in 293T cells and analysed the mRNA levels of lysosomal genes. RT-PCR data show that lysosomal gene expression was upregulated by C-ETS2 (Figure 8(a)). We then downregulated C-ETS2 in 293T and SHY5Y cells and found that lysosomal gene expression was remarkably decreased (Figures 8(b) and 8(c)). Finally, we repeated the experiment in primary neurons and obtained results consistent with our hypothesis: C-ETS2 knockdown reduced lysosomal gene expression; although lysosomal gene expression in the control cells increased under oxidative stress, it increased only slightly after C-ETS2 downregulation under oxidative stress (Figure 8(d)).

## 4. Discussion

In conclusion, we verified that oxidative stress can induce cell apoptosis and that lysosomes help cells cope with oxidative stress. We found lysosomal gene expression to be increased under oxidative stress in cells and in the brains of MPTP-treated mice and that the upregulation of lysosomal genes is regulated by the transcription factor TFEB. We also explored TFEB's role in cell survival under oxidative stress. The results show that TFEB knockdown in cells increases cell apoptosis under oxidative stress; conversely, overexpression of TFEB protects cells from apoptosis. Correspondingly, we also found that TFEB overexpression in the SN reduces DA neuronal loss in MPTP-treated mice. TFEB expression is increased, and it is translocated to the nucleus by oxidative stress. Finally, we demonstrate that C-ETS2 mediates TFEB expression and further regulates the upregulation of lysosomal genes under oxidative stress.

Oxidative stress is a shared feature of neurodegenerative diseases (e.g., Alzheimer's disease and Parkinson's disease). High levels of oxygen utilization and ATP synthesis in the brain render this organ more vulnerable to oxidative stress than some other tissues. The excessive reactive oxygen species generated as an organism ages may result in oxidative damage to proteins, lipids, and DNA and may result in neuronal apoptosis [4]. In this study, we used hydrogen peroxide treatment as a model for oxidative stress and confirmed that this treatment increases apoptosis in primary neurons. Recently, several reports have noted that autophagy can be induced by oxidative stress to help maintain cellular homeostasis. During the final stages of autophagy, lysosomes play an important role in the bulk degradation of aggregated proteins. In 2003, Bendiske and Bahr showed that the upregulation of lysosomal activity by PADK leads to clearance of accumulated proteins and restores microtubule integrity in AD-slice models [28]. Dehay et al. also reported that the genetic or pharmacological induction of lysosomal biogenesis attenuates AP accumulation and dopaminergic cell death in PD models that also suffer from oxidative stress [30]. Here, we confirm that lysosomal function is necessary for cells to maintain resistance to oxidative stress. It has been reported that lysosome hydrolase activity levels are enhanced in the hippocampus with the onset of early-stage Alzheimer's disease and that lysosomal vacuole quantities are increased in several protein-deposition disorders [28]. However, these studies did not clearly examine the mechanisms of lysosomal function enhancement. Accordingly, our study investigated the molecular mechanisms that result in enhanced lysosomal function. We measured lysosomal gene expression in the SHY5Y cell line, the primary neurons, and the brains of mice under oxidative stress and found that the mRNA and protein levels of lysosomal genes are upregulated by oxidative stress, which may account for the lysosomal activation induced by oxidative stress.

In 2009, Sardiello et al. discovered that TFEB, acting as a key transcription factor of lysosome biogenesis, positively regulates lysosomal gene expression. TFEB overexpression in cultured cells induces lysosome biogenesis and stimulates the clearance of aggregated molecules [9]. Therefore, we speculated that TFEB perhaps mediates the increased expression of lysosomal genes. We knocked down TFEB in cells and found that lysosomal gene expression declined under oxidative stress. These results show that TFEB mediates the regulation of lysosomal genes under oxidative stress. Then, we further explored how TFEB is regulated by oxidative stress. After analysing TFEB mRNA and protein levels, we found TFEB expression to be increased under oxidative stress. At the same time, we found that TFEB translocates to the nucleus following exposure to hydrogen peroxide. Recent studies have reported that the localization of TFEB is affected by its phosphorylation status, which is regulated by ERK or mTORC1, and we found that levels of phosphorylated TFEB in the nucleus also increased in conjunction with total TFEB protein levels.

We then examined the role of TFEB in cell survival under oxidative stress. The results show that cell survival increases following TFEB overexpression and, conversely, that TFEB knockdown leads to cell apoptosis under oxidative stress. These results confirm the protective role of TFEB in promoting cell survival in response to oxidative stress. We also overexpressed TFEB in the SN of the MPTP-induced oxidative stress model and found that TFEB protects the dopaminergic neurons of the SN. This is also consistent with recent reports that TFEB overexpression effectively blocks the development *α*-syn-induced pathology while protecting nigral DA neurons in rats with Parkinson's disease [14].

We show here that TFEB transcription is activated by oxidative stress, and we also found that C-ETS2 mediates TFEB activity under oxidative stress. We then found that C-ETS2 is indeed involved in the transcriptional regulation of lysosomal genes and TFEB in response to oxidative stress. Thus, we conclude that C-ETS2 mediates the TFEB expression pathway, protects cells from apoptosis, and may act as a compensatory response that supports resistance to oxidative stress. This result is also in agreement with a study that reported that C-ETS2 is activated by oxidative stress and accounts for the observations of glial survival via induced Bcl-xL expression [22].

It is noteworthy that lysosomal genes were found to be upregulated only under stimulation by moderate oxidative stress. Their expression was not affected or even downregulated when cells were treated with high concentrations of hydrogen peroxide or for long periods of time. We assume that, during the early stages of oxidative stress, lysosomal functions may be activated by oxidative stress and may help cells survive. However, long-term or severe oxidative stress may result in lysosomal damage and hydrolase leakage, resulting in immediate cell death. Several studies have also found loss in lysosomal function during amyloidogenic processes and the aggregation of *α*-synuclein [28]. The C-ETS2-mediated TFEB-induction mechanism may play a central role in the response of neurons to oxidative stress during the early stages of neurodegenerative disease, thus revealing a new avenue of neuroprotective therapy in neurodegenerative diseases.

## Figures and Tables

**Table 1:** shRNA.

shRNA	Sequence
*Tfeb*-mouse-shRNA	5′-CGGCAGTACTATGACTATGAT-3′
*TFEB*-homo-shRNA	5′-GAGACGAAGGTTCAACATCAA-3′
*C*-*ets2*-mouse-shRNA	5′-CAAGAAAAGACAGAAGACCAA-3′
*C*-*ETS2*-homo-shRNA	5′-GCGGCAGGATGAATGATTTCG-3′

**Table 2:** RT primers.

Gene	Sequence
*C*-*ETS2*-F RT(H)	5′-CCCCTGTGGCTAACAGTTACA-3′
*C*-*ETS2*-R RT(H)	5′-AGGTAGCTTTTAAGGCTTGACTC-3′
*C*-*ets2*-F RT(M)	5′-CCTGTCGCCAACAGTTTTCG-3′
*C*-*ets2*-R RT(M)	5′-GGAGTGTCTGATCTTCACTGAGA-3′
*ARSA*-F RT(H)	5′-AGAGCTTTGCAGAGCGTTCAG-3′
*ARSA*-R RT(H)	5′-ATACGCATGGTCTCAGGTCCA-3′
*Arsa*-F RT(M)	5′-CTGGGGACCCTCTTTTTGGC-3′
*Arsa*-R RT(M)	5′-AACTGGGGTGCCCATAGGA-3′
*ARSB*-F RT(H)	5′-ATCAGTGAAGGAAGCCCATCC-3′
*ARSB*-R RT(H)	5′-ACACGGTGAAGAGTCCACGAA-3′
*Arsb*-F RT(M)	5′-CCACGGGCTCTGGAACAAC-3′
*Arsb*-R RT(M)	5′-GGTGTCCTTCACTGATTGTCTTC-3′
*ATP6V0E1*-F RT(H)	5′-CATTGTGATGAGCGTGTTCTGG-3′
*ATP6V0E1*-R RT(H)	5′-AACTCCCCGGTTAGGACCCTTA-3′
*Atp6v0e1*-F RT(M)	5′-GCATACCACGGCCTTACTGT-3′
*Atp6v0e1*-R RT(M)	5′-TGATAACTCCCCGGTTAGGAC-3′
*ATP6V1H*-F RT(H)	5′-GGAAGTGTCAGATGATCCCCA-3′
*ATP6V1H*-R RT(H)	5′-CCGTTTGCCTCGTGGATAAT-3′
*Atp6v1h*-F RT(M)	5′-CCAAGATGGACATTCGAGGTG-3′
*Atp6v1h*-R RT(M)	5′-CACTTTGTTGGCACGAACTTC-3′
*CLCN7*-F RT(H)	5′-TGATCTCCACGTTCACCCTGA-3′
*CLCN7*-R RT(H)	5′-TCTCCGAGTCAAACCTTCCGA-3′
*Clcn7*-F RT(M)	5′-CGCCAGTCTCATTCTGCACT-3′
*Clcn7*-R RT(M)	5′-GAGGATCGACTTCCGGGTC-3′
*CTSB*-F RT(H)	5′-AGTGGAGAATGGCACACCCTA-3′
*CTSB*-R RT(H)	5′-AAGAAGCCATTGTCACCCCA-3′
*Ctsb*-F RT(M)	5′-CAGGCTGGACGCAACTTCTAC-3′
*Ctsb*-R RT(M)	5′-TCACCGAACGCAACCCTTC-3′
*CTSD*-F RT(H)	5′-AACTGCTGGACATCGCTTGCT-3′
*CTSD*-R RT(H)	5′-CATTCTTCACGTAGGTGCTGGA-3′
*Ctsd*-F RT(M)	5′-GCTTCCGGTCTTTGACAACCT-3′
*Ctsd*-R RT(M)	5′-CACCAAGCATTAGTTCTCCTCC-3′
*GALNS*-F RT(H)	5′-TTGTCGGCAAGTGGCATCT-3′
*GALNS*-R RT(H)	5′-CCAAACCACTCATCAAATCCG-3′
*Galns*-F RT(M)	5′-TCATGGACGATATGGGGTGG-3′
*Galns*-R RT(M)	5′-AGATGGTGAGCACAAAGGGTT-3′
*GBA*-F RT(H)	5′-TGGGTACCCGGATGATGTTA-3′
*GBA*-R RT(H)	5′-AGATGCTGCTGCTCTCAACA-3′
*Gba*-F RT(M)	5′-GCCAGGCTCATCGGATTCTTC-3′
*Gba*-R RT(M)	5′-GAGTGCTCTCGTAACGGCT-3′
*GLA*-F RT(H)	5′-AGCCAGATTCCTGCATCAGTG-3′
*GLA*-R RT(H)	5′-ATAACCTGCATCCTTCCAGCC-3′
*Gla*-F RT(M)	5′-TGGCGCGGACTCCTACTAT-3′
*Gla*-R RT(M)	5′-GCCATCTGCATGAACAGTTGC-3′
*GNS*-F RT(H)	5′-CCCATTTTGAGAGGTGCCAGT-3′
*GNS*-R RT(H)	5′-TGACGTTACGGCCTTCTCCTT-3′
*Gns*-F RT(M)	5′-GGCATGACGCCACTGAAGAA-3′
*Gns*-R RT(M)	5′-GGGCACATAGGCGCTAGAG-3′
*HEXA*-F RT(H)	5′-CAACCAACACATTCTTCTCCA-3′
*HEXA*-R RT(H)	5′-CGCTATCGTGACCTGCTTTT-3′
*Hexa*-F RT(M)	5′-TGGCCCCAGTACATCCAAAC-3′
*Hexa*-R RT(M)	5′-GGTTACGGTAGCGTCGAAAGG-3′
*LAMP1*-F RT(H)	5′-ACGTTACAGCGTCCAGCTCAT-3′
*LAMP1*-R RT(H)	5′-TCTTTGGAGCTCGCATTGG-3′
*Lamp1*-F RT(M)	5′-CAGCACTCTTTGAGGTGAAAAAC-3′
*Lamp1*-R RT(M)	5′-CCATTCGCAGTCTCGTAGGTG-3′
*LAMP2*-F RT(H)	5′-GGGTTCAGCCTTTCAATGTG-3′
*LAMP2*-R RT(H)	5′-GTTGACCAGTATTGCATGTTG-3′
*Lamp2*-F RT(M)	5′-TGTATTTGGCTAATGGCTCAGC-3′
*Lamp2*-R RT(M)	5′-ACCCACTGCAACAGGAATAAG-3′
*TFEB*-F RT(H)	5′-AGGCTGTCATGCATTACATGCA-3′
*TFEB*-R RT(H)	5′-CTTGTTCCCATAGGTCTCGGA-3′
*Ttfeb*-F RT(M)	5′-AAGGTTCGGGAGTATCTGTCTG-3′
*Ttfeb*-R RT(M)	5′-GGGTTGGAGCTGATATGTAGCA-3′
GAPDH-F RT	5′-TGACAACGAATTTGGCTACA-3′
GAPDH-R RT	5′-GTGGTCCAGGGGTCTTACTC-3′
*L32*-F RT(M)	5′-TCTGGTGAAGCCCAAGATCG-3′
*L32*-R RT(M)	5′-CTCTGGGTTTCCGCCAGTT-3′
